# Determining depression and related factors in a society affected by COVID-19 pandemic

**DOI:** 10.1177/0020764020938807

**Published:** 2020-06-30

**Authors:** Gonca Ustun

**Affiliations:** Department of Psychiatric Nursing, Faculty of Health Sciences, Amasya University, Amasya, Turkey

**Keywords:** COVID-19, coronavirus, pandemic, quarantine, depression

## Abstract

**Background:**

Turkey has one of the highest death rates in the world due to COVID-19 pandemic. The pandemic caused anxiety and depression in individuals. However, there is insufficient information on the effects of COVID-19 on individuals and their coping methods. Therefore, mental problems associated with the pandemic need to be evaluated rapidly.

**Aims:**

This study was carried out to determine depression levels and related factors in a society affected by COVID-19.

**Method:**

The study was planned using a descriptive cross-sectional design. The study started 2 weeks after the first diagnosis of COVID-19 in Turkey and was carried out between March 23 and April 3, 2020. The study included 1115 adult participants who were between 18 and 65 years of age and were citizens of the Turkish Republic. The study was carried out using online questionnaires, and data were collected with the Personal Information Form and the Beck Depression Inventory. The data were evaluated using IBM SPSS Statistics, version 20 software program.

**Results:**

The depression scores of female participants who were between 18 and 29 years of age, single, students, and had less income than their expenses were found to be higher than others. People who experienced fear of being infected and infecting others, had a cleaning obsession, anxiety about the future, sadness, and anxiousness experienced depression at lower levels when compared to other participants. Participants who had to change their place of residence during the quarantine, experienced loneliness, fear of death, hopelessness, sleep problems, felt useless and worthless, started to smoke and drink alcohol, and experienced depression at moderate levels. Depression scores of those who spent time with their family, made time for themselves, were busy with home education or work were lower compared to others.

**Conclusion:**

The COVID-19 pandemic caused mild-level depression in the Turkish society.

## Introduction

The coronavirus causes upper respiratory infection which presents with symptoms such as high fever and difficulty in breathing (Gralinski & Menachery, 2020). The new coronavirus (COVID-19) was first detected in November 2019 in Wuhan in the Hubei state of China (World Health Organization (WHO), 2020b). It spread rapidly within China and to other countries (Shigemura et al., 2020).

Since January 23, 2020, strict quarantine measures have been taken in Wuhan and other regions of China to prevent the COVID-19 pandemic (J. B. Li et al., 2020). The reopening of the schools after spring break was postponed, citizens were encouraged to work from their homes, to stay at home if possible, to use personal protective equipment such as face masks, and all meetings were canceled (Kickbusch & Leung, 2020). On January 30, 2020, the WHO (2020b) declared the COVID-19 outbreak to be a Public Health Emergency of International Concern, a pandemic, on March 11, 2020. As of writing this article, the number of positive COVID-19 cases is 7,255,960 and the number of deaths due to this fatal virus have reached 412,583 (WHO, 2020b). The highest number of cases were observed in the United States of America, Brazil, Russian Federation, the United Kingdom, and India, in that order (WHO, 2020a).

The first COVID-19 case in Turkey was detected on March 10, 2020, and the first death due the virus was reported on March 17, 2020 (Ministry of Health, 2020). In Turkey, teams have been created to conduct studies on the COVID-19, and preventive measures have been taken that are required for the pandemic plan. As of March 16, 2020, flexible working hours and a home office working system have been adopted throughout Turkey. On the same date, schools and universities started distance education. A lockdown was imposed on individuals above 65 years of age as of March 21, 2020. As of April 4, 2020, a lockdown was also applied on individuals under 20 years of age, and entrance/exit restrictions to 30 metropolises and the city Zonguldak. As of writing this article, the number of cases in Turkey was declared to be 173,036 and the number of deaths, 4,746 (Ministry of Health, 2020). Turkey has one of the highest death rates in the world, being the 11th country in order of death rates (WHO, 2020a).

Administration of the quarantine measures taken to combat the pandemic affects many aspects of life at the individual and societal levels (Qiu et al., 2020). The COVID-19 pandemic not only threatens human safety and physical health but also causes mental problems in societies affected by this situation by causing thousands of deaths worldwide (W. Li et al., 2020; Liu et al., 2020; Zhou, 2020). A series of undesirable emotional and behavioral problems developed in individuals during this period: an increase in negative emotions such as despair, anxiety, guilt, stigma, insomnia, anger, fear of being infected, an increase in alcohol consumption and smoking, and social isolation (Dong & Bouey, 2020; Ho et al., 2020; J. B. Li et al., 2020; W. Li et al., 2020; Shigemura et al., 2020; Wang et al., 2020; Zhou, 2020).

The pandemic caused anxiety disorders such as somatization, post-traumatic stress disorder, and panic disorder and depression in individuals (Ho et al., 2020; J. B. Li et al., 2020; Qiu et al., 2020; Shigemura et al., 2020). However, there is insufficient information on the effects of COVID-19 on individuals and their coping methods (Ho et al., 2020; W. Li et al., 2020). Therefore, mental problems associated with the pandemic need to be evaluated rapidly (Gao et al., 2020).

This study in Turkey applies to adult individuals between 18 and 65 years of age: it is structured to be a lead study for future studies and for assessing depression in individuals who overcome this period after the effect of the pandemic in Turkey ends. Moreover, considering this pandemic as a social crisis, this study is important in terms of realizing the seriousness of the conditions and demonstrating its effects on the society. This study will also approach subjects such as implementing strengthening programs and developing coping skills by determining the groups most affected by the pandemic.

This study was carried out to determine depression levels and related factors in a society affected by COVID-19.

## Method

### Study design

This study was planned using the descriptive cross-sectional design, a quantitative research method.

### Time and place of the study

The study was carried out between March 23 and April 3, 2020. It started 2 weeks after the first confirmed diagnosis of COVID-19 in Turkey and was completed within 12 days in seven regions.

### Population and sample

The random opportunistic online sample was determined for this study representing the Turkish society. All individuals who met the inclusion criteria and answered the questions completely were included in the study sample. Inclusion criteria were as follows:

- Being a citizen of Turkish Republic.
- Being an adult between 18 and 65 years of age.

Exclusion criteria were as follows:

- Receiving treatment at the hospital during the time that the study was in progress.
- Having been diagnosed with COVID-19.
- Having been diagnosed with psychiatric illness.

The study was completed with 1115 participants; 44 of the 1159 original participants were excluded from the study for reason of having a psychiatric illness diagnosis.

### Data collection tools

The study data were collected using a Personal Information Form that was developed by the researcher and the Beck Depression Inventory (BDI).

Personal Information Form: the Personal Information Form includes closed-ended questions that include participants’ sociodemographic characteristics, their personal, family-related, social, educational, or word-related problems, mental disorders that they experienced due to the COVID-19 pandemic, and how they handled the “quarantine/lockdown” process initiated to prevent the pandemic from spreading further.

BDI: the Turkish validity and reliability study of the inventory developed by Beck (1961) was carried out by Hisli (1989). The BDI is a 21-item self-evaluation scale that measures depression symptoms. Every item is scored from 0 to 3; possible scores range from 0 to 63. It was suggested that those who obtain 17 and more points are in the group with a risk for depression (Hisli, 1989). Depression levels were defined as follows: 0–9, minimal; 10–16, mild; 17–29, medium; 30–63, severe (Kilinc & Torun, 2011). The Cronbach’s alpha value of Hisli’s validity and reliability study was .74, that of this study was .90.

### Study process

Data collection forms were opened for access through Google Forms over the internet as online questionnaires using social media channels. Announcements open for sharing were made via Instagram, Facebook, WhatsApp, LinkedIn, and Twitter accounts, and messages were sent for further spread of the announcements. The participants filled out the forms in approximately 10 minutes.

The first COVID-19 case in Turkey was diagnosed on March 11, 2020. Administration of the questionnaire continued for 12 days between the 13th and 24th days of the pandemic and was completed when the number of valid questionnaires reached 1115. The duration of the study was planned according to the quarantine measures of the Ministry of Health. During the 12-day period during which the administration of the questionnaire continued, a lockdown was imposed on individuals over 65 years of age in addition to precautions such as closing workplaces, schools, and universities, making an effort to obey the lockdown, and minimizing human contact. Entry/exit restrictions to 30 metropolitan areas and the Zonguldak municipality and a face mask obligation in markets and bazaars were implemented; a lockdown was imposed on individuals under the age of 20 in April 4, 2020, in addition to the precautions already in place.

Because the most recent residence- and age-related measures would have affected the sample group, the study was concluded on April 3, 2020. The response rate of the questionnaires, and the speed of questionnaires reaching the potential audience, decreased, which led to the discontinuing of administration of the questionnaire.

### Ethical considerations

The Ministry of Health (consent code: T17_25_46) and the Amasya University Clinical Research Ethics Committee (consent number: 15,386,878-044) granted permission for the study. Participants were provided with an obligatory informed consent form before they accessed the questionnaire forms.

### Data analysis

The data were evaluated using the IBM SPSS Statistics, version 20 software program. Descriptive statistics were calculated for the classification of study data and explanation of their characteristics. Because the variables were not normally distributed, Mann–Whitney *U*-test was used for the comparison of two groups, and the Kruskal–Wallis H test was used for the comparison of three or more groups. For the evaluation of the results, the significance level was accepted as 0.05: *p* < .05 indicated a significant difference, and *p* > .05 indicated no significant difference. When there was a significant difference between more than two groups, multiple comparison was carried out using the Mann–Whitney U-test to determine the source of the significance. The significance level 0.05 was determined to be the comparison threshold.

## Results

**Figure 1.**
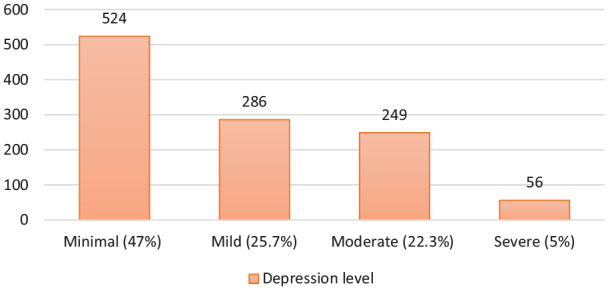
Distribution of participants regarding their depression levels.

The mean age of the 1115 participant was 27.98 ± 8.79 years (min. = 18, max. = 65), 71.7% of the participants were female, 62.9% were single, 69.9% had graduated from university, 42.7% were students, and 26.9% were public employees (Table 1). Participants’ mean depression score was 12.07 ± 9.60 (min. = 0.00, max. = 63); therefore, the overall depression was at the mild level. Among the participants, 47% showed minimal-level depression symptoms (0–9 points); 25.7%, mild-level depression symptoms (10–16 points); 22.3%, moderate-level depression symptoms (17–29 points); and 5%, severe depression symptoms (30–63 points) (Figure 1).

**Table 1.** Comparison of participants’ sociodemographic characteristics and depression.

Variables	*n*	%	BDI	Significance		
	1115	100	Mean ± *SD*	Mean rank	Test	*p*
Age groups						
18–29 years of age	693	62.2	13.13 ± 10.07	592.07	*χ*^2^ = 22.366 Multiple comparisons (1–2) (1–3) (1–4)	*p* = **.000**
30–39 years of age	291	26.1	10.54 ± 8.69	509.34		
40–49 years of age	103	9.2	10.24 ± 8.04	502.88		
50–65 years of age	28	2.5	8.25 ± 7.80	423.20		
Gender						
Female	799	71.7	12.98 ± 9.81	590.75	*Z* = −5.404	*p* = **.000**
Male	316	28.3	9.75 ± 8.62	475.20		
Education level						
Primary school and secondary school	50	4.5	11.98 ± 9.54	556.72	*χ*^2^ = 11.544 Multiple comparisons (2–4) (3–4)	*p* = **.009**
High school	131	11.7	12.48 ± 9.49	577.85		
University	779	69.9	12.45 ± 9.74	570.84		
Postgraduate	155	13.9	9.79 ± 8.70	477.08		
Profession						
Student	476	42.7	13.68 ± 10.34	608.30	*χ*^2^ = 23.939 Multiple comparisons (1–2)	*p* = **.000**
Public employee	300	26.9	10.11 ± 8.32	495.50		
Private sector employee	150	13.5	11.65 ± 9.97	535.94		
Own business	45	4.0	11.37 ± 8.97	539.46		
Unemployed	110	9.9	11.55 ± 8.38	554.30		
Other	34	3.0	11.23 ± 8.84	539.10		
Income status						
Income lower than expenses	270	24.2	15.37 ± 10.84	661.89	*χ*^2^ = 38.018 Multiple comparisons (1–2) (1–3)	*p* = **.000**
Equal income and expenses	590	52.9	11.14 ± 8.80	531.61		
Income higher than expenses	255	22.9	10.72 ± 9.18	509.05		
Region of residence						
Mediterranean region	66	5.9	12.34 ± 9.06	581.87	*χ*^2^ = 14.150 Multiple comparisons (2–4) (4–6) (4–7)	*p* = **.028**
East Anatolia	46	4.1	10.28 ± 9.89	476.77		
Aegean region	67	6.0	12.14 ± 8.81	572.12		
Southeastern Anatolia	55	4.9	16.30 ± 11.50	692.50		
Central Anatolia	175	15.7	12.65 ± 10.49	566.99		
Black Sea region	508	45.6	11.66 ± 9.04	550.00		
Marmara region	198	17.8	11.70 ± 9.73	539.37		
Place of residence						
City center	653	58.6	11.77 ± 9.26	550.78	*χ*^2^ = 0.905	*p* = .636
County town	357	32.0	12.48 ± 10.26	565.48		
Village	105	9.4	12.51 ± 9.33	577.44		
Marital status						
Single	701	62.9	12.88 ± 10.09	581.83	*Z* = −3.218	*p* = **.001**
Married	414	37.1	10.68 ± 8.52	517.65		
Individuals participants are living with						
Alone	75	6.7	9.60 ± 8.59	469.85	*χ*^2^ = 6.431	*p* = .092
Nuclear family (single partner/partner and children)	772	69.2	11.97 ± 9.20	560.90		
Extended family (partner, children, and family elders)	149	13.4	13.30 ± 11.29	578.21		
Other	119	10.7	12.68 ± 10.20	569.46		
Individuals over 65 years of age for whom the participants should provide care or with whom they live together						
Yes	181	16.2	12.40 ± 9.62	570.81	*Z* = −0.585	*p* = .558
No	934	83.8	12.00 ± 9.59	555.52		

BDI: Beck Depression Inventory. Bold values denote statistical significance at the *p* < 0.05 level.

Table 1 shows the comparison of participants’ sociodemographic characteristics and depression scores. In an analysis of participants’ gender and marital status, those who were female and single had significantly higher depression levels than other participants (*p* < .05). There was a significant difference between participants’ depression scores in terms of their age groups, education status, occupation, income levels, and place of residence (*p* < .05). The depression levels were higher in the 18–29 year age group than in other groups, in students than in public employees, in individuals who had income lower than their expenses than among other participants. Participants who had completed an undergraduate education had significantly lower levels of depression than those who had graduated from high school or university. No significant difference was found between depression levels in terms of participants’ place of residence, people they were living with, and participants’ family members over 65 years of age who needed care (*p* > .05).

Table 2 shows the comparison of participants’ COVID-19–related characteristics and depression scores. No significant difference was found between depression scores in terms the following characteristics: having a chronic illness determined by the Ministry of Health to be within the risk group; taking a COVID-19 test; whether a family member took a COVID-19 test or having been diagnosed with the disease; to what extent respondents said they pay attention to and follow the warnings of the Ministry of Health; the information sources; and the person with whom the participants go through this process (*p* > .05). Participants who said that they need psychological support during the quarantine had significantly higher depression levels than others (*p* < .05). It was found that the participants who took the COVID-19 test and reported that they need psychological support during the quarantine showed moderate-level depression symptoms and were in the risk group for depression.

**Table 2.** Comparison of participants’ COVID-19 characteristics and depression scores.

Variables		*N*	%	BDI	Significance		
		1115	100	Mean ± *SD*	Mean rank	Test	*p*
Do you have a chronic illness that the Ministry of Health determined for the risk groups?							
Yes		101	9.1	12.55 ± 8.94	588.37	*Z* = −0.995	*p* = .320
No		1014	90.9	12.02 ± 9.66	554.97		
Did you take a test after you had a suspicion of COVID-19?							
Yes		3	0.3	23.66 ± 26.10	693.50	*Z* = −0.730	*p* = .465
No		1112	99.7	12.03 ± 9.52	557.63		
Did a family member of yours take a test after they had a suspicion of COVID-19?							
Yes		34	3.0	13.82 ± 8.27	646.07	*Z* = −1.621	*p* = .105
No		1081	97.0	12.01 ± 9.63	555.23		
Was a family member of your diagnosed with COVID-19?							
Yes		12	1.1	12.83 ± 8.00	610.92	*Z* = −0.573	*p* = .567
No		1103	98.9	12.06 ± 9.61	557.42		
To what extent do you pay attention to and apply COVID-19 pandemic warnings?							
I do not pay attention to them at all		3	0.3	15.66 ± 5.85	753.33	*χ*^2^ = 5.375	*p* = .146
I partly pay attention to them		42	3.8	15.66 ± 13.88	612.40		
I pay attention to them		452	40.5	11.34 ± 9.15	535.41		
I pay attention to them too much		618	55.4	12.33 ± 9.53	569.87		
With whom do you frequently spend the quarantine period?							
Alone		84	7.5	11.02 ± 8.87	528.63	*χ*^2^ = 2.073	*p* = .557
Family		962	86.3	12.12 ± 9.50	561.99		
Friend		24	2.2	11.45 ± 12.01	486.10		
Colleague		45	4.0	13.13 ± 11.59	565.77		
Do think you need psychological support during this period?							
Yes		278	24.9	19.30 ± 10.81	786.13	*Z* = −13.645	*p* = **.000**
No		837	75.1	9.66 ± 7.80	482.23		
Which resources do you use to obtain information about COVID-19^a^							
I do not follow the agenda	Yes	151	13.5	11.74 ± 10.74	529.22	*Z* = −1.182	*p* = .237
	No	964	86.5	12.12 ± 9.41	562.51		
Television and internet news	Yes	774	69.4	12.27 ± 9.68	564.92	*Z* = −1.082	*p* = .279
	No	341	30.6	11.60 ± 9.39	542.29		
Social media	Yes	544	48.8	12.64 ± 10.25	570.67	*Z* = −1.284	*p* = .199
	No	571	51.2	11.52 ± 8.90	545.93		
Ministries and government agencies	Yes	770	69.1	11.88 ± 9.54	551.00	*Z* = −1.085	*p* = .278
	No	345	30.9	12.48 ± 9.72	573.62		
Healthcare professionals	Yes	261	23.4	12.50 ± 10.63	559.61	*Z* = −0.092	*p* = .926
	No	854	76.6	11.93 ± 9.26	557.51		
Scientific researches	Yes	239	21.4	11.10 ± 9.22	522.08	*Z* = −1.947	*p* = .052
	No	876	78.6	12.33 ± 9.68	567.80		
Friends and relatives	Yes	197	17.7	12.48 ± 10.13	567.95	*Z* = −0.478	*p* = .632
	No	918	82.3	11.98 ± 9.48	555.86		

BDI: Beck Depression Inventory.

Bold values denote statistical significance at the *p* < 0.05 level.

a Multiple options were marked and the percentages were calculated over the sample size.

Of the participants, 86.3% went through this period accompanied by their families; 55.4% claimed to pay too much attention to the COVID-19 pandemic warnings; 69.4% used television and internet news; and 69.1% used the resources of the ministries and government agencies for obtaining information about the COVID-19 pandemic (Table 2).

Table 3 shows the problems that participants frequently encountered as a result of the pandemic and their mean depression scores. In analyzing the personal problems of participants, those who reported that their daily routine and cleaning habits changed, and that they were exposed to social media more than previously had significantly higher depression scores than others (*p* < .05).

**Table 3.** Comparison of participants’ problems during the quarantine and their depression scores.

Variables		*n*	%	BDI	Significance		
		1115	100	Mean ± *SD*	Mean rank	Test	*p*
Situation that personally affected you the most^a^							
I was not affected at all	Yes	93	8.3	6.79 ± 8.21	343.40	*Z* = −6.718	*p* = **.000**
	No	1022	91.7	12.55 ± 9.57	577.53		
My daily routines (such as meals, sleep, shopping) have changed	Yes	521	46.7	14.05 ± 10.36	622.00	*Z* = −6.220	*p* = **.000**
	No	594	53.3	10.32 ± 8.50	501.87		
My cleaning habits (such as washing hands, cleaning the house) have changed	Yes	647	58.0	12.76 ± 9.69	584.96	*Z* = −3.290	*p* = **.001**
	No	468	42.0	11.11 ± 9.38	520.73		
I was exposed to social media more than before	Yes	538	48.3	13.37 ± 9.75	607.74	*Z* = −4.985	*p* = **.000**
	No	577	51.7	10.85 ± 9.30	511.62		
Situation in the family and social life that affected you the most^a^							
I was not affected at all	Yes	93	8.3	6.50 ± 7.46	339.78	*Z* = −6.831	*p* = **.000**
	No	1022	91.7	12.57 ± 9.61	577.86		
I have had to stay away from my family	Yes	147	13.2	14.55 ± 10.87	629.65	*Z* = −2.898	*p* = **.004**
	No	968	86.8	11.69 ± 9.34	547.12		
Meetings with my friends, relatives, and neighbors have become rare	Yes	574	51.5	12.41 ± 9.53	573.41	*Z* *=−*1.647	*p* = .100
	No	541	48.5	11.70 ± 9.66	541.65		
I drift apart from by entertainment and social life	Yes	734	65.8	12.78 ± 10.03	579.35	*Z* = −3.075	*p* = **.002**
	No	381	34.2	10.69 ± 8.53	516.87		
I have had to change my place of residence	Yes	150	13.5	17.06 ± 11.48	705.18	*Z* = −6.022	*p* = **.000**
	No	965	86.5	11.29 ± 9.03	535.12		
I got lonely	Yes	60	5.4	17.33 ± 11.16	713.22	*Z* = −3.842	*p* = **.000**
	No	1055	94.6	11.77 ± 9.42	549.17		
Situation in your work/education life that affected you the most^a^							
I was not affected at all	Yes	161	14.4	9.54 ± 8.98	460.11	*Z* = −4.173	*p* = **.000**
	No	954	85.6	12.49 ± 9.64	574.52		
I drifted apart from my school/work place	Yes	642	57.6	12.44 ± 9.85	569.11	*Z* = −1.343	*p* = .179
	No	473	42.4	11.56 ± 9.23	542.93		
My financial income has been negatively affected	Yes	159	14.3	15.39 ± 9.62	680.76	*Z* = −5.196	*p* = **.000**
	No	956	85.7	11.51 ± 9.48	537.58		
I had to go to work	Yes	193	17.3	12.16 ± 9.08	570.18	*Z* = −0.579	*p* = .563
	No	922	82.7	12.05 ± 9.70	555.45		
I had to close down my business	Yes	27	2.4	15.14 ± 8.47	694.02	*Z* = −2.224	*p* = **.026**
	No	1088	97.6	11.99 ± 9.61	554.62		
I had to work from home	Yes	159	14.3	10.50 ± 8.69	507.03	*Z* = −2.157	*p* = **.031**
	No	956	85.7	12.33 ± 9.72	566.48		
Situation that affected your mental health the most^a^							
I was not affected at all	Yes	100	9.0	5.28 ± 6.94	284.37	*Z* = −8.914	*p* = **.000**
	No	1015	91.0	12.73 ± 9.56	584.96		
I was afraid to be infected by the virus	Yes	611	54.8	13.36 ± 9.79	606.72	*Z* = −5.567	*p* = **.000**
	No	504	45.2	10.49 ± 9.12	498.94		
I was afraid to infect others with the virus	Yes	508	45.6	13.41 ± 9.87	607.32	*Z* = −4.682	*p* = **.000**
	No	607	54.4	10.94 ± 9.21	516.72		
I experienced fear of death	Yes	203	18.2	18.78 ± 10.94	770.16	*Z* = −10.388	*p* = **.000**
	No	912	81.8	10.57 ± 8.59	510.78		
I have started to experience cleaning obsession	Yes	360	32.3	14.88 ± 10.36	653.93	*Z* = −6.875	*p* = **.000**
	No	755	67.7	10.72 ± 8.91	512.26		
I felt useless and worthless	Yes	48	4.3	24.39 ± 11.61	894.59	*Z* = −7.409	*p* = **.000**
	No	1067	95.7	11.51 ± 9.12	542.86		
I was worried about my future	Yes	486	43.6	15.29 ± 10.09	671.85	*Z* = −10.386	*p* = **.000**
	No	629	56.4	9.58 ± 8.39	470.03		
I was unhappy	Yes	498	44.7	14.13 ± 10.22	627.35	*Z* = −6.466	*p* = **.000**
	No	617	55.3	10.40 ± 8.72	502.03		
I experienced anxiety	Yes	500	44.8	15.45 ± 9.99	678.62	*Z* = −11.287	*p* = **.000**
	No	615	55.2	9.31 ± 8.31	459.93		
I experienced hopelessness	Yes	352	31.6	17.70 ± 10.07	749.54	*Z* = −13.502	*p* = **.000**
	No	763	68.4	9.47 ± 8.16	469.63		
I experienced sleep problems	Yes	216	19.4	19.68 ± 9.91	810.22	*Z* = −12.831	*p* = **.000**
	No	899	80.6	10.24 ± 8.57	497.40		
I started to smoke and use alcohol	Yes	24	2.2	26.20 ± 10.85	937.60	*Z* = −5.843	*p* = **.000**
	No	1091	97.8	11.75 ± 9.33	549.65		

BDI: Beck Depression Inventory.

Bold values denote statistical significance at the *p* < 0.05 level.

a Multiple options were marked and the percentages were calculated over the sample size.

In analyzing the situations affecting participants’ family relationships and social life, participants who had to be away from their family, place of residence, entertainment, and social life, and those who reported that they felt lonely during this process, had significantly higher depression scores than others (*p* < .05). Participants who had to change their place of residence and were lonely were in the risk group and had moderate-level depression (Table 3).

In the analysis of situations affecting participants’ work and educational life, those who had to close their business and reported that their financial income was affected negatively had significantly higher depression scores than others (*p* < .05). Participants who could not go to school or had to work from home had significantly lower depression scores (*p* *<* .05). In comparing the depression scores of those who had to be away from their school or work and had to go to work with others, no significant difference was found (*p* *>* .05; Table 3).

There was a significant difference between the participants’ depression scores and their psychological problems experienced during this period (*p* *<* .05). The participants who were afraid of being infected by the virus or infecting others, were obsessed with cleaning, had anxiety about the future, were unhappy and anxious, and were found to have mild-level depression scores. The depression levels of participants who experienced fear of death, feeling useless and worthless, hopelessness, sleep problems, and started to smoke and consume alcohol were found to be at a moderate level (Table 3).

In analyzing the situations participants are frequently affected during the quarantine, 58% of them reported that their cleaning habits changed, 65.8% that they were feeling deprived of their entertainment and social life, and 57.6% said that they were deprived of their school and workplace. Moreover, 54.8% of the participants reported that they experienced fear of being infected by the virus, and 45.6% that they experienced fear of infecting others with the virus (Table 3).

Table 4 shows the comparison of participants’ coping strategies (behaviors that helped them to feel better during the quarantine) and their depression scores. The most helpful methods for the participants were spending time with their families (56.5%) and making more time for themselves (51.6%). Participants who spent time with their families, made time for themselves, and were busy with home education or work were found to have significantly lower depression scores than others (*p* < .05). Participants who used social media, communicated with their friends through phone calls or video calls, and cleaned their houses more often had significantly higher depression scores than others (*p* < .05). It was found that behaviors such as going on a walk, exercising, sleeping regularly, eating a balanced diet, and praying created no significant difference in depression scores (*p* > .05); however, participants who applied those behaviors had lower depression levels than others.

**Table 4.** Comparison of participants’ coping methods during the quarantine and their depression scores.

Variables		*n*	%	BDI	Significance		
		1115	100	Mean ± *SD*	Mean rank	Test	*p*
Coping strategies during the quarantine period^a^							
Sparing time for myself (reading book, watching movies, etc.)	Yes	575	51.6	11.26 ± 9.13	531.08	*Z* = −2.883	*p* = **.004**
	No	540	48.4	12.93 ± 10.00	586.67		
Spending time with my family	Yes	630	56.5	10.92 ± 8.46	527.34	*Z* = −3.626	*p* = **.000**
	No	485	43.5	13.56 ± 10.72	597.82		
Spending time on social media	Yes	317	28.4	13.98 ± 9.68	630.15	*Z* = −4.719	*p* = **.000**
	No	798	71.6	11.31 ± 9.46	529.34		
Talking to my friends through phone call or video call	Yes	276	24.8	14.09 ± 11.39	606.68	*Z* = −2.898	*p* = **.004**
	No	839	75.2	11.40 ± 8.83	541.99		
Working (doing my job/studying)	Yes	279	25.0	10.58 ± 8.82	508.90	*Z* = −2.944	*p* = **.003**
	No	836	75.0	12.56 ± 9.79	574.39		
Cleaning the house	Yes	336	30.1	13.46 ± 10.32	602.30	*Z* = −3.020	*p* = **.003**
	No	779	69.9	11.47 ± 9.21	538.89		
Going on a walk in open areas	Yes	58	5.2	11.44 ± 10.29	519.22	*Z* = −0.943	*p* = .346
	No	1057	94.8	12.10 ± 9.56	560.13		
Exercising	Yes	112	10.0	10.43 ± 7.78	517.24	*Z* = −1.413	*p* = .158
	No	1003	90.0	12.25 ± 9.76	562.55		
Sleeping regularly	Yes	187	16.8	11.28 ± 10.28	519.06	*Z* = −1.814	*p* = .070
	No	928	83.2	12.22 ± 9.45	565.85		
Eating regularly	Yes	201	18.0	11.63 ± 9.72	540.59	*Z* = −0.847	*p* = .397
	No	914	82.0	12.16 ± 9.57	561.83		
Praying	Yes	332	29.8	11.86 ± 9.12	556.76	*Z* = −0.084	*p* = .933
	No	783	70.2	12.15 ± 9.79	558.53		

BDI: Beck Depression Inventory.

Bold values denote statistical significance at the *p* < 0.05 level.

a Multiple options were marked and the percentages were calculated over the sample size.

## Discussion

The COVID-19 pandemic caused psychological problems in China and other societies that were similarly affected (J. B. Li et al., 2020; Xiao, 2020). One of the first studies that determined the psychological problems of China’s general population was carried out by Qiu et al. (2020). It collected 52,730 valid questionnaires from 36 provinces, self-governing territories, and municipalities, Hong Kong, Macau, and Taiwan. Of the individuals participating in the questionnaire, 35% experienced psychological problems. In the study of Wang et al. (2020), 53.8% of the general population of China regarded the psychological effect of the pandemic to be moderate or severe.

Administration of strict quarantine measures never previously experienced caused people to gradually drift apart from one another and caused isolation in the society (Qiu et al., 2020; Xiao, 2020). Lack of interpersonal communication can also cause or worsen depression (Xiao, 2020). In studies evaluating a society’s overall mental health in terms of COVID-19 through online questionnaires in 31 provinces and self-governing territories of China, individuals above 18 years of age experienced depression (Gao et al., 2020; Liu et al., 2020). In the study by Gao et al. (2020), carried out with 4,872 participants, 48.3% showed depression symptoms. In the study of Liu et al. (2020) of the 14,592 participants, 53.5% showed depression symptoms.

In the study carried out with 1210 participants in 194 cities in China using online questionnaires, 69.7% of the participants were found to be normal, 13.8% had mild-level depression, 12.2% had moderate-level depression, and 4.3% had severe- or extreme-level depression (Wang et al., 2020). In this study, with participants from all regions of Turkey, participants’ mean depression scores were at a mild level: 47% of the participants showed minimal-level depression symptoms, 25.7% mild-level depression symptoms, 22.3% moderate-level depression symptoms, and 5% severe-level depression symptoms.

In different studies carried out during the pandemic, certain sociodemographic characteristics affected depression levels. Similarly to the current study, these studies also determined that being female, a student (Wang et al., 2020), and young and single (Liu et al., 2020) are risk factors for depression. Females were more inclined to depression, which may have inclined them to be more affected by the pandemic. Although being away from school might seem to be an advantage for students, it can be concluded that in an education system that is unfamiliar and the uncertainty of the process in that system may have caused depression symptoms. Because younger and single individuals use social media more than others, they often obtain excessive and inaccurate information that may have triggered depression.

Trusting in the measures taken by the government and trying to adapt better encourage the society to work together support the struggle against the pandemic (Ho et al., 2020). In Italy, 3,452 young adults above 18 years of age reported being cautious about health measures recommended for countering the COVID-19 pandemic. Approximately 100% of the participants approved four public health measures: 95.2% avoided hand shaking, 94.7% avoided social communities, 93.1% followed lockdown for nonobligatory activities, and 89% approved closing non-mandatory shops (Barari et al., 2020). In this study, 95.9% of the participants reported that they pay attention to and apply the measures recommended by the Ministry of Health. Participants who followed the recommendations had lower depression points than the ones who did not.

Insufficient information and tabloid news about COVID-19 caused the anxiety and fear against the situation to increase (Jiloha, 2020; J. B. Li et al., 2020; Shigemura et al., 2020). The WHO emphasized that people should minimize their habit of following news about COVID-19, and that they should obtain information only once or twice daily during certain hours and only from reliable sources. In addition, following the WHO (2020c) website and local health authority platforms is also recommended to distinguish accurate from inaccurate information. Of the participants, 69.1% obtained information from the Ministry and government agencies. However, people who obtained information from social media constituted 48.8% of the study; this group had the highest depression score as compared to other groups.

It is important for individuals to maintain their daily routines, regular life activities, and join entertaining activities for coping with their personal problems during the quarantine (Jiloha, 2020; Park & Park, 2020). Depression scores of the participants who stated that their daily routine and cleaning habits changed and were exposed to social media more were higher compared to those of others. In a study conducted in the China, more than 80% of the participants stated that they were exposed to social media more than before and that was positively correlated with their anxiety level (Gao et al., 2020).

Minimizing the contact among people to prevent spreading the virus requires a conscious effort. In contagious diseases, including COVID-19, social distancing should be minded even in groups that show no symptoms and are not at risk. People should avoid public places and stay at home. They should leave 1 m distance from other people when they are outdoors (Jiloha, 2020). Despite social distance warnings, staying in touch with family, friends, and colleagues is recommended as coping methods (Park & Park, 2020). This study determined that those who were alone during the quarantine were in the risk group for depression and had moderate-level depression symptoms. However, those who communicated with their friends through voice or video calls were found to be more depressive. This situation may have arisen because people experiencing psychological problems want to get in touch with others more frequently.

Because closing public services and the collapse of the industries negatively affected the economy, many people experienced financial losses and were exposed to a risk of unemployment. This situation intensified the negative emotions individuals experienced (Ho et al., 2020). People who stated that they had to close their businesses and their financial income was negatively affected had higher depression scores, but depression scores of the people who did not go to work and had to work from home were found to be lower. Because staying at home decreased infection risk, provided a safer environment than the workplace, and provided the opportunity to spend more time with their families, working from home can be seen as an advantage.

Different studies evaluating mental effects of the COVID-19 pandemic on society found that people often experienced anxiety, despair, fear of being infected, worry, hopelessness, and sleep deprivation (Dong & Bouey, 2020; Gao et al., 2020; Ho et al., 2020; W. Li et al., 2020; Liu et al., 2020; Park & Park, 2020; Shigemura et al., 2020; Wang et al., 2020). In this study, people who experienced fear of being infected and infecting others, had a cleaning obsession, anxiety about the future, sadness, and anxiousness were found to have mild-level depression symptoms. Depression levels of participants who experienced fear of death, feeling useless and worthless, hopelessness, sleep problems, and started to smoke and consume alcohol were found to be at moderate level.

### Limitations

This cross-sectional study had some limitations. Because the sample size was large, the questionnaires were carried out on the internet to make rapid evaluation possible. Therefore, access to elderly population (50–65 year of age) was limited (2.5% of the sample) and for the same reason, participation of younger individuals was higher (62.2% of the sample). Because the study was conducted in the Black Sea region of Turkey, participation from this region was higher (45.6% of the sample). Moreover, the sample was limited only to those who used social media; therefore, homogeneity of regions could not be achieved.

In this study, numerous parameters such as depression could have been evaluated with nonmeasurable variables. However, only a single variable was evaluated considering that the questionnaires were administrated online; the number of questions was planned so that the participants would not have difficulties in terms of time; and the access to internet could be limited. The study was carried out during the early periods of the quarantine process. Therefore, because there was no control group, it was uncertain whether the variables were affected by the quarantine process or by previously diagnosed depression.

## Conclusion and recommendations

In Turkey, the COVID-19 pandemic caused mild-level depression in the society. The depression scores of female participants who were between 18 and 29 years of age, single, students, and had less income than their expenses were found to be higher than others. People who experienced fear of being infected and infecting others, had a cleaning obsession, anxiety about the future, sadness, and anxiousness experienced depression at lower levels when compared to other participants. Participants who had to change their place of residence during the quarantine, experienced loneliness, fear of death, hopelessness, sleep problems, felt useless and worthless, and started to smoke and drink alcohol experienced depression at moderate levels. Depression scores of those who spent time with their family, made time for themselves, were busy with home education or work were lower compared to others.

Early intervention programs and studies that will strengthen the society’s mental health should be carried out in groups at risk for depression. Studies analyzing effects of this period on mental health in different groups, defining the needs and problems of quarantined people should be carried out. Studies reevaluating the depression in individuals who overcame this period should be carried out after the pandemic ends.
